# Greenhouse gas emissions from U.S. crude oil pipeline accidents: 1968 to 2020

**DOI:** 10.1038/s41597-023-02478-4

**Published:** 2023-08-24

**Authors:** Hongfang Lu, Zhao-Dong Xu, Kaihui Song, Y. Frank Cheng, Shaohua Dong, Hongyuan Fang, Haoyan Peng, Yun Fu, Dongmin Xi, Zizhe Han, Xinmeng Jiang, Yao-Rong Dong, Panpan Gai, Zhiwei Shan, Yuli Shan

**Affiliations:** 1 China-Pakistan Belt and Road Joint Laboratory on Smart Disaster Prevention of Major Infrastructures, Southeast University, Nanjing, 210096 China; 2 Data-Driven EnviroLab, School of Public Policy, University of North Carolina at Chapel Hill, Chapel Hill, NC 27599 USA; 3 Department of Mechanical Engineering, University of Calgary, Calgary, Alberta T2N 1N4 Canada; 4 School of Safety and Ocean Engineering, China University of Petroleum (Beijing), Beijing, 102249 China; 5 Yellow River Laboratory, Zhengzhou University, Zhengzhou, 450001 China; 6 School of Civil Engineering, Xi’an University of Architecture and Technology, Xi’an, 710055 China; 7 School of Civil Engineering and Mechanics, Jiangsu University, Zhenjiang, 212013 China; 8 School of Geography, Earth and Environmental Sciences, University of Birmingham, Birmingham, B15 2TT UK

**Keywords:** Atmospheric science, Energy infrastructure

## Abstract

Crude oil pipelines are considered as the lifelines of energy industry. However, accidents of the pipelines can lead to severe public health and environmental concerns, in which greenhouse gas (GHG) emissions, primarily methane, are frequently overlooked. While previous studies examined fugitive emissions in normal operation of crude oil pipelines, emissions resulting from accidents were typically managed separately and were therefore not included in the emission account of oil systems. To bridge this knowledge gap, we employed a bottom-up approach to conducted the first-ever inventory of GHG emissions resulting from crude oil pipeline accidents in the United States at the state level from 1968 to 2020, and leveraged Monte Carlo simulation to estimate the associated uncertainties. Our results reveal that GHG emissions from accidents in gathering pipelines (~720,000 tCO_2_e) exceed those from transmission pipelines (~290,000 tCO_2_e), although significantly more accidents have occurred in transmission pipelines (6883 cases) than gathering pipelines (773 cases). Texas accounted for over 40% of total accident-related GHG emissions nationwide. Our study contributes to enhanced accuracy of the GHG account associated with crude oil transport and implementing the data-driven climate mitigation strategies.

## Background & Summary

The United States (U.S.) possesses an extensive network of crude oil pipelines, with the total length over 130,000 kilometers by the end of 2022^1,2^. These pipelines, due to their immense scale and connectivity, play an essential role in facilitating energy supplies and economic growth of the country^3^. However, occurrence of accidents within this intricate pipeline system can pose significant environmental challenges, particularly cause release of substantial amount of greenhouse gases (GHGs)^4,5^. Methane is the primary GHG released from crude oil pipelines when accidents occur. It has 28–36 times of global warming potential (GWP) compared to CO_2_ for 100-year time span, and 84–87 times of GWP over a 20-year timeframe (GWP_20_)^6^. Despite high GWP, these unintended emissions are usually neglected in official estimates of national GHG emissions from bottom-up approaches^7,8^, as emissions resulting from accidents are typically managed separately and their impacts on climate change are usually ignored when public health risks present. The accidents, although occurring occasionally, can release tons of GHG emissions^9^, undermining the global climate mitigation efforts if not properly addressed^10,11^. Quantifying emissions resulting from large-scale crude oil pipeline accidents is therefore of utmost importance, as it serves as a fundamental component for better understanding the broader implications of climate change mitigation efforts and developing robust response strategies to minimize the adverse impacts of these accidents for both researchers and policymakers^12–14^.

Previous studies pertaining to quantifying upstream GHG emissions from crude oil have predominantly utilized lifecycle assessment (LCA) and centred on major procedures, including production, transportation, and refining processes^15–19^. These “well-to-wheels” life-cycle GHG emissions from petroleum amount to ~1.7 gigatons of CO_2_ equivalent (GtCO_2_e), representing ~5% of total fuel combustion emissions^15^. In the refining process, well-to-refinery carbon intensity ranges from ~1.5 to 46.9 gCO_2_e/MJ^16^. The carbon intensity range of refining is 13.9–62.1 kg CO_2_ equivalent per barrel^17^. When studying the GHG emissions from each process, only macroscopic factors (such as distance travelled and throughput) under normal pipeline operations are carefully studied. As a common practice, studies employing LCA to analyze carbon intensity of crude oil production often ignore impacts that are difficult to measure or less well understood^15^. Despite the existing research on carbon emissions in the oil supply chain, the carbon emissions resulting from accidents have not been fully defined. It should be noted that fugitive emissions are fundamentally different from emissions caused by accidents. Fugitive emissions only consider leaks that occur during normal operations and do not include emissions resulting from unexpected accidents^20,21^. For instance, valve leaks during the regular operation of pipelines would be classified as fugitive emissions, whereas the explosion of the Nord Stream pipeline in September 2022 was classified as accident-related emissions^9^.

Recent studies have recognized the necessity of considering natural gas leaks from oil and gas pipelines in city and national climate actions^11,22^. However, these studies mainly rely on methane monitoring devices, and the process is labor-intensive and usually occurs in individual observation points. The recent movement of employing satellites to remote sense the methane content^23^ offers a top-down approach to estimate and address the potential underestimation from traditional bottom-up approaches; however, it usually cannot identify the detailed sources of methane emissions and therefore becomes limited in developing effective strategies for policymakers. While understanding the implications for conducting climate change-focused integrity management is of paramount importance, the GHG emissions resulting from crude oil pipeline accidents have been largely unexplored in previous studies.

To fill this gap, this study quantifies the GHG emissions resulting from crude oil pipeline accidents in the U.S. from 1968 to 2020. The accidents include both gathering and transmission pipeline systems, considering the combustion/explosion conditions associated with the accidents. In comparison to the accident dataset previously provided by the Pipeline and Hazardous Materials Safety Administration (PHMSA), the dataset proposed in this study (1) quantifies previously unaccounted GHG emissions in accident records, and (2) incorporates uncertainties involved in GHG emission assessment. Consequently, this study develops an accurate inventory of GHG emissions resulting from crude oil pipeline accidents. This work contributes to rectifying the omissions within the official audits of the oil system, thereby enhancing the accuracy of GHG emissions accounting. Furthermore, by attaining the GHG emissions associated with each crude oil pipeline accident, operators can utilize this information to conduct more profound climate change-oriented risk assessments across various regions, design parameters, and operational conditions of pipelines. This, in turn, facilitates the formulation of pragmatic and actionable measures to mitigate the occurrence of accidents and potentially offers guidance for future pipeline designs. Additionally, the proposed inventory methodology may serve as a reference for investigating GHG emissions in similar accidents.

This study yields three distinct datasets, including GHG emissions resulting from oil pipeline accidents assessed by year (1968 to 2020), GHG emissions resulting from oil pipeline accidents assessed by state, and GHG emissions from single-point accidents. The specific descriptions of the three datasets are presented in the section entitled “Data Records”. Each dataset carries significant implications: (1) The accurate assessment of GHG emissions resulting from crude oil pipeline accidents enables decision-makers to acquire a more precise understanding of the consequential impact on climate change. Consequently, this comprehensive knowledge fosters informed decision-making in the realm of subnational climate actions and policy formulation. (2) The datasets we provide will serve as a valuable resource for pipeline operators, providing them enhanced insight into the potential GHG emission risks arising from accidents. By making them access to the valuable information, operators are able to develop proactive measures and effective counterstrategies, thereby bolstering their social standing and reputation^19^.

## Methods

### Data collection

We collected data required to quantify GHG emissions resulting from crude oil pipeline accidents, such as the volume of oil spills and accident conditions. The PHMSA has documented data of pipeline accidents involving hazardous liquid in the U.S. since 1968. Hazardous liquids include biofuels, carbon dioxide, crude oil, highly volatile liquids, and refined petroleum products^24^. The PHMSA requires all operators to report the occurrence of an accident within 30 days in accordance with Title 49 of the Code of Federal Regulations (49 CFR Parts 191, 195)^25,26^ (see Supplementary Note 1 for the definition of crude oil pipeline accidents). We extracted accident information from all crude oil pipelines, including gathering pipelines and transmission pipelines. However, various changes and updates in the PHMSA’s data management pose challenges to time-series analysis. Different levels of details are documented in four distinct time periods: 1968 to 1985, 1986 to 2001, 2002 to 2009, and 2010 to the present, with the most comprehensive information available for the period from 2010 to the present. Table 1 presents detailed information on accidents contained in each period of the dataset.

**Table 1:** Information on crude oil pipeline accidents contained in the PHMSA dataset.

Category	1968 to 1985	1986 to 2001	2002 to 2009	2010 to the present
Operator information	Included	Included	Included	Included
Accident information	Included	Included	Included	Included
Release information	Included	Included	Included	Included
Consequence information	Included	Included	Included	Included
Pipeline design information	Included	Included	Included	Included
Pipeline maintenance information	Included but limited	Included but limited	Included	Included
Accident cause	Included	Included	Included	Included
Drug and alcohol testing information	Not included	Not included	Included	Included
Number of items	61	63	259	654
Number of accidents recorded	2488 (transmission pipelines); 276 (gathering pipelines)	1093 (transmission pipelines); 234 (gathering pipelines)	1270 (transmission pipelines); 105 (gathering pipelines)	Continuously updated (2032 accidents as of 2020 for transmission pipelines, 158 accidents as of 2020 for gathering pipelines)

Regardless of the period, the PHMSA provides detailed information about when and where accidents happened and the volume of crude oil leaked. However, it is still insufficient for assessing the impact of the accident on climate change. First, while the PHMSA recorded the volume of oil spills for each accident, it did not provide information regarding GHG emissions associated with each incident. Second, crucial information on oil recovery for transmission pipelines is missing from 1968 to 1985, which is essential for accounting for GHG emissions in both spill events and emergent responses.

### Calculating GHG emissions from single-point accidents

**Fig. 1:**
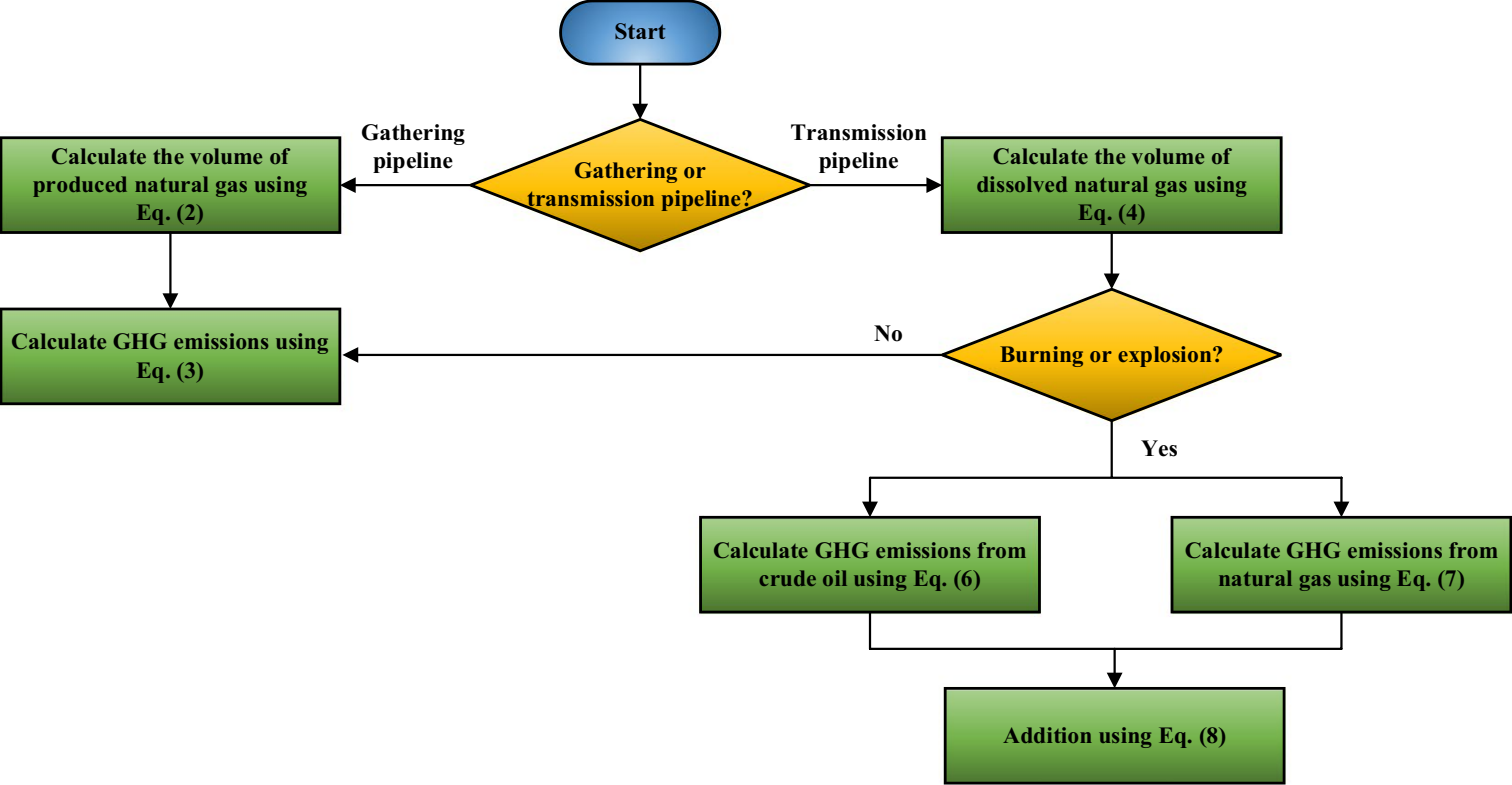
Flow chart for calculation of GHG emissions from crude oil pipeline accidents.

Leaks in oil pipelines release GHGs (methane). However, the GHG emissions associated with oil spills vary depending on oil products being transported in pipelines and accident conditions (such as non-combustion, combustion, and explosion). We designed a workflow to illustrate how the GHG emissions for single-point accidents were calculated, as shown in Fig. 1. We further analyzed GHG emissions from crude oil pipelines in two categories according to their functions^27,28^, namely gathering pipelines and transmission pipelines (details about the two types of pipelines are shown in Supplementary Table 1).

In gathering pipelines, a considerable amount of natural gas is often present as a byproduct during the process of crude oil extraction at the wellhead. It is imperative to calculate the GHG emissions resulting from pipeline accidents by considering the production gas-oil ratio (PGOR) and conditions whether combustion/explosion occurs. PGOR refers to the ratio of natural gas to crude oil produced from oil wells in the process of oil field exploitation^29^. It can be derived from historical production data in the U.S., as shown in Eq. (1).

1 $$\begin{array}{c}PGOR=\frac{{p}_{g}}{{p}_{co}}\end{array}$$

where, PGOR is production gas-oil ratio, cubic feet per barrel (ft^3^/bbl); *p*_*g*_ is production rate of natural gas, ft^3^/day; and *p*_*co*_ is production rate of crude oil, bbls/day. The volume of natural gas released can be deduced by applying Eq. (2).

2 $$\begin{array}{c}{V}_{gas,gathering}={V}_{crudeoil,gathering}\times {\rm{PGOR}}\end{array}$$

where *V*_*gas,gathering*_ is the volume of natural gas released from gathering pipelines, ft^3^; and *V*_*crude oil,gathering*_ is the volume of oil spills from gathering pipelines, bbls.

From accident data recorded by the PHMSA spanning from 1968 to 2020, it was found that gathering pipelines have not experienced combustion/explosion accidents. This suggests that the GHG emissions from gathering pipeline accidents are solely due to the release of natural gas resulting from crude oil spills. If combustion or explosion is not involved in the accident, the GHG emissions from the release of natural gas can be calculated by Eq. (3)^30^.

3 $$\begin{array}{c}{{\rm{GHG}}}_{gas,unburned}=2.8\times 1{0}^{-5}\times {{\rm{GWP}}}_{{{\rm{CH}}}_{4}}\times {\rho }_{gas}\times {V}_{gas,gathering}\end{array}$$

where *GHG*_*gas,unburned*_ is GHG emissions from the release of unburned natural gas, metric tons of carbon dioxide equivalent (tCO_2_e); *ρ*_*gas*_ is natural gas density, kg/m^3^; and ${{\rm{GWP}}}_{{{\rm{CH}}}_{4}}$ is the global warming potential of methane, ${{\rm{GWP}}}_{{{\rm{CH}}}_{4}}=27.9$ under the 100-year timeframe from IPCC the Sixth Assessment Report (AR6) from Working Group I (WRI)^31^.

Removing natural gas from the oil-gas fluid during the processing stage yields higher-quality crude oil, which is transported to refineries via transmission pipelines. However, natural gas could still present in transmission pipelines, albeit in small quantities^32^. In the case of pipeline accidents, pipeline pressure rapidly decreases, leading to escape of a limited amount of dissolved natural gas from the crude oil. The quantity of natural gas emissions can be calculated by considering the dissolved gas-oil ratio (DGOR)^32^, as shown by Eq. (4). The DGOR refers to the ratio between the volume of dissolved natural gas in crude oil under a specific pressure condition, as described by Eq. (5).

4 $$\begin{array}{c}{V}_{gas,transmission}={V}_{crudeoil,transmission}\times {\rm{DGOR}}\end{array}$$

where *V*_*g,t*_ is release volume of natural gas from transmission pipelines, ft^3^; *V*_*co*_ is oil spills from transmission pipelines, bbls; and DGOR is dissolved gas-oil ratio, ft^3^/bbl.

5 $$\begin{array}{c}{\rm{DGOR}}={\gamma }_{gas}{\left[\frac{P}{18}\times \frac{1{0}^{0.0125{\rm{API}}}}{1{0}^{0.00091T}}\right]}^{1.2048}\end{array}$$

where *γ*_*gas*_ is gas-specific gravity; API is oil-API gravity; *P* is absolute pressure, psi; and *T* is temperature, °F. *P* is determined according to the accident pressure provided in the PHMSA database. *T* is uniformly taken as 60°F^32^. API values vary widely across the U.S.

According to data provided by PHMSA, transmission pipelines are susceptible to combustion or explosion accidents. If there is combustion or explosion in the crude oil transmission pipeline accident, the GHG emissions caused by the crude oil can be calculated according to Eq. (6).

6 $$\begin{array}{c}{{\rm{GHG}}}_{crudeoil,burned}=0.43\times {V}_{crudeoil,transmission}\times {f}_{u}\end{array}$$

where *GHG*_*crude oil, burned*_ is GHG emissions produced by the accident (with combustion or explosion) for crude oil transmission pipeline, tCO_2_e; *V*_*crude oil, transmission*_ is the volume of crude oil spilled from the transmission pipeline, bbls; and *f*_*u*_ is the unrecovered fraction following crude oil leakage, percent. Note that after crude oil spills, the escaped natural gas is proportional to the amount of oil spilled. However, a portion of the crude oil can be recovered through various methods^33^–^39^. If combustion or explosion occurs during the accident, the recovered crude oil is not involved in the combustion accident and does not contribute to GHG emissions. Moreover, in the scenario where transmission pipelines are involved in combustion or explosion accidents, the GHG emissions generated from the combustion of the released natural gas can be computed utilizing Eq. (7)^30^.

7 $$\begin{array}{c}{{\rm{GHG}}}_{gas,burned}=5.48\times 1{0}^{-5}\times {V}_{gas,transmission,burned}\times {f}_{c}\times {{\rm{GWP}}}_{{{\rm{CO}}}_{2}}\end{array}$$

where *GHG*_*gas,burned*_ is GHG emissions from natural gas combustion or explosion, tCO_2_e; *V*_*gas,transmission, burned*_ is the volume of natural gas that burns or explodes from transmission pipelines, ft^3^; *f*_*c*_ is the fraction of natural gas oxidized into carbon dioxide^40^; and ${{\rm{GWP}}}_{{{\rm{CO}}}_{2}}$ is the global warming potential of carbon dioxide, ${{\rm{GWP}}}_{{{\rm{CO}}}_{2}}=1$ under the 100-year timeframe from IPCC AR6 WGI report. Therefore, the total GHG emissions resulting from a transmission pipeline accident involving combustion or explosion are:

8 $$\begin{array}{c}{{\rm{GHG}}}_{total,burned}={{\rm{GHG}}}_{gas,burned}+{{\rm{GHG}}}_{crudeoil,burned}\end{array}$$

where GHG_total, burned_ is the total GHG emissions from transmission pipeline accident (with combustion or explosion).

### Monte Carlo simulation for GHG emission inventory

Monte Carlo simulation constitutes a numerical methodology grounded in stochastic sampling and statistical analysis, finding extensive utility in addressing the intricacies of uncertainty analysis within complex systems. Within the context of GHG emission estimation, the salient advantage of Monte Carlo simulation resides in its capacity to adeptly capture interdependencies among multiple uncertain parameters, thereby affording a comprehensive and precise assessment of uncertainties. By iteratively sampling from the probability distributions of each parameter, Monte Carlo simulation engenders the generation of a profusion of potential scenarios, thereby encompassing a spectrum of prospective outcomes inherent to the emissions process^41,42^. In this study, the GHG emissions from gathering and transmission pipeline accidents are investigated separately. For the gathering pipeline system, a total of 773 accidents from 1968 to 2020 are recorded, and the spill volumes of these accidents can be obtained from PHMSA. Based on the probability density function (PDF) of PGOR, 773 data points are generated to correspond to the PGOR values of these accidents, and the gas volume is calculated based on Eq. (2). Additionally, when estimating GHG emissions according to Eq. (3), the density of natural gas is randomly selected between 0.66 and 1.05 kg/m^3^. The entire process is repeated 200,000 times to obtain the uncertainty of GHG emission inventory for gathering pipeline accidents.

From 1968 to 2020, a total of 6,883 transmission pipeline accidents were recorded. When estimating the dissolved natural gas associated with these accidents based on Eqs. (4) and (5), the values of *γ*_*gas*_ and API are uncertain. *γ*_*gas*_ is randomly selected between 0.55 and 0.87, and a PDF of [23 + WEIB(10.8, 6.35)] is used to generate 6,883 data points corresponding to the API values of these accidents. Additionally, the involvement of combustion and explosion in accidents needs to be considered. If combustion or explosion occurs, the dissolved natural gas and GHG emissions from the burned crude oil are estimated according to Eq. (7) and Eq. (6) respectively. It is important to note that, of the 222 accidents involving combustion or explosion, only 97 accidents record volumes of recovered crude oil, while the volumes of burned crude oil remain unknown for the remaining 125 accidents. Therefore, a PDF of [−0.001 + EXPO(0.417)] fitted based on the unrecovered ratio from these 97 accidents is used to generate 125 data points corresponding to the *f*_*u*_ values of these 125 accidents. The entire process is repeated 200,000 times to estimate the uncertainty of GHG emission inventory for transmission pipeline accidents. Furthermore, the data utilized in the Monte Carlo simulation are sourced from Supplementary Table 2, as indicated.

## Data Records

Three datasets were generated in this study:

1. GHG emissions resulting from gathering and transmission pipeline accidents annually from 1968 to 2020, as presented in Figshare^43^.
2. GHG emissions resulting from gathering and transmission pipeline accidents in different states during the period from 1968 to 2020, as shown in Figshare^43^. The gathering pipeline dataset covers a total of 27 states, while the transmission pipeline dataset includes 44 states.
3. GHG emissions from a total of 7,656 individual accidents that occurred from 1968 to 2020, as provided in Figshare^43^.

It is important to note that the first two datasets display the mean values and uncertainties obtained from 200,000 Monte Carlo simulations, whereas the third dataset presents the mean value along with the upper and lower bounds of the 90% confidence interval derived from the same 200,000 Monte Carlo simulations.

**Fig. 2:**
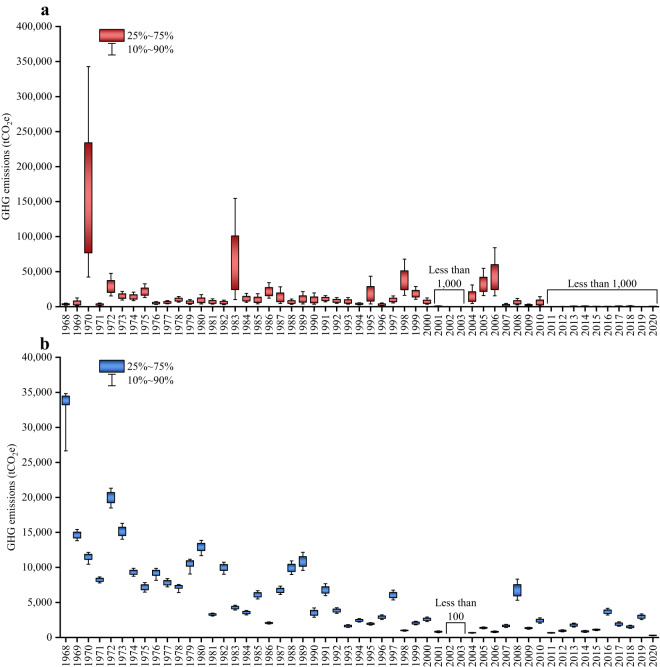
Annual GHG emissions caused by crude oil pipeline accidents from 1968 to 2020. (**a**) Gathering pipelines. (**b**) Transmission pipelines. Note that the annotations “less than 1,000” and “less than 100” in subfigures a and b refer to the average values derived from Monte Carlo simulations. Specific data simulated by Monte Carlo are available at Figshare^43^.

Figure 2 illustrates that gathering pipelines were associated with the highest GHG emissions in the year 1970, amounting to (170,119 ± 123,398) tCO_2_e. The subsequent years with notable emissions were 1983 and 2006, with values of (70,491 ± 59,660) and (45,179 ± 27,998) tCO_2_e, respectively. Additionally, the GHG emissions (average values from Monte Carlo simulations) for the years 2001 to 2003 and 2011 to 2020 remained below 1,000 tCO_2_e. For transmission pipelines, the year 1968 exhibited the highest GHG emissions, amounting to (32,464 ± 4,252) tCO_2_e. This was followed by the years 1972 and 1969, with emissions of (19,928 ± 1,128) and (14,610 ± 636) tCO_2_e, respectively. In comparison between the gathering and transmission systems, although the number of accidents in the gathering pipelines (773) is significantly lower than that in the transmission pipelines (6,883), the GHG emissions resulting from accidents (~720,000 tCO_2_e) are higher in the gathering pipelines than in the transmission pipelines (~290,000 tCO_2_e).

**Fig. 3:**
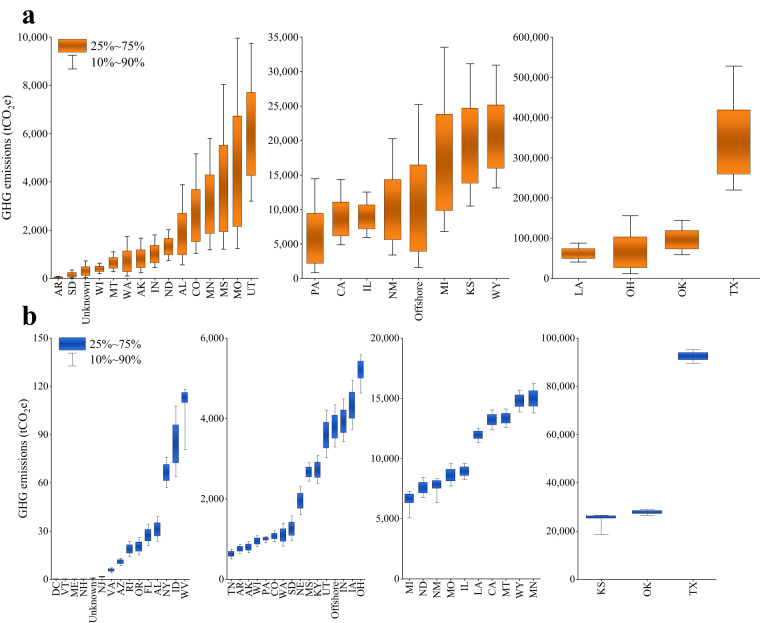
GHG emissions caused by crude oil pipeline accidents in various states. (**a**) Gathering pipelines. (**b**) Transmission pipelines. Specific data simulated by Monte Carlo are available at Figshare^43^. The heat map of GHG emissions is presented in Supplementary Fig. 1. “Unknown” indicates that the PHMSA dataset does not specify the state where the incident occurred. The full names of the state abbreviations are shown in ref. ^46^.

Figure 3 highlights that, in the gathering pipeline system, Texas, Oklahoma, and Ohio have the highest GHG emissions. Texas, in particular, has the GHG emissions up to (352,675 ± 126,164) tCO_2_e, accounting for nearly half of the emissions within the gathering pipeline system. In the transmission pipeline system, Texas again has the highest GHG emissions, amounting to (92,496 ± 2,247) tCO_2_e. Following Texas, Oklahoma and Kansas rank the second and the third, with emissions of (27,795 ± 937) and (24,298 ± 4,243) tCO_2_e, respectively.

**Fig. 4:**
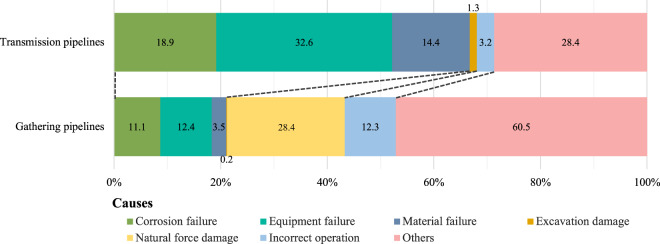
Percentage of GHG emissions from various causes. (**a**) Gathering pipelines. (**b**) Transmission pipelines. The proportions are calculated based on the average values obtained from Monte Carlo simulations. The category “Others” mainly includes the following ten specific reasons: (1) destruction caused by boats, barges, drilling rigs, or other maritime equipment or vessels that are set adrift or have lost their mooring; (2) damage caused by cars, trucks, or other motorized vehicles or equipment not involved in excavation; (3) electrical arcing originating from other equipment or facilities; (4) intentional acts of damage; (5) accidents categorized as miscellaneous; (6) occurrence of nearby industrial, man-made, or other fire/explosion as the primary cause of the accident; (7) damage resulting from other external forces; (8) pre-existing mechanical damage unrelated to excavation; (9) accidents arising from routine or normal fishing or other maritime activities not related to excavation; (10) occurrences with unknown causes.

The PHMSA categorizes the causes of pipeline accidents as corrosion, equipment failure, material failure, excavation damage, incorrect operation, natural force damage, and others^44,45^. Figure 4 shows that equipment failure and incorrect operation are the primary factors contributing to GHG emissions from gathering pipeline accidents, excluding the “others” category. In the transmission pipeline system, the equipment failure is the primary contributor to GHG emissions, accounting for 32.6% of the total.

## Technical Validation

### Uncertainties

For gathering pipelines, uncertainties in estimating GHG emissions from accidents primarily stem from the values of PGOR and natural gas density. In transmission pipelines, uncertainties arise from DGOR, natural gas density, API of crude oil, the combustion ratio of crude oil in combustion accidents, and the conversion ratio of natural gas. We employed Monte Carlo simulations (n = 200,000 times) to obtain the uncertainties using standard deviation. Ref. ^43^ presents the uncertainties in GHG emissions inventory on an annual basis, indicating that the uncertainty range for gathering pipelines varies from ± 28.9% to ± 84.6%, while for transmission pipelines, it ranges from ± 4.1% to ± 16.8%. The uncertainties in GHG emissions inventory for gathering pipelines in different states, ranging from ± 30.1% to ± 86.5%. The uncertainties in GHG emissions inventory for transmission pipelines in various states, ranging from ± 2.4% to ± 19.7%.

### Validation of the estimation method

To validate the reliability of the proposed methodology and generated datasets, a deterministic approach was employed to estimate GHG emissions resulting from accidents. The results were then compared with those obtained through Monte Carlo simulations. In the deterministic approach, all parameters were set as fixed values, as indicated in Supplementary Table 3. The years 2010 to 2020 were selected for validation purposes since PHMSA provides more detailed records of accidents during this period. Furthermore, Eq. (9) was employed to assess the deviation between the deterministic method and the average values obtained from Monte Carlo simulations.

9 $$\begin{array}{c}E=\frac{{V}_{D}-{V}_{M}}{{V}_{D}}\end{array}$$

where *E* is the deviation; *V*_*D*_ is GHG emissions from the deterministic methods, tCO_2_e; and *V*_*M*_ is the average GHG emissions from Monte Carlo simulations, tCO_2_e.

**Fig. 5:**
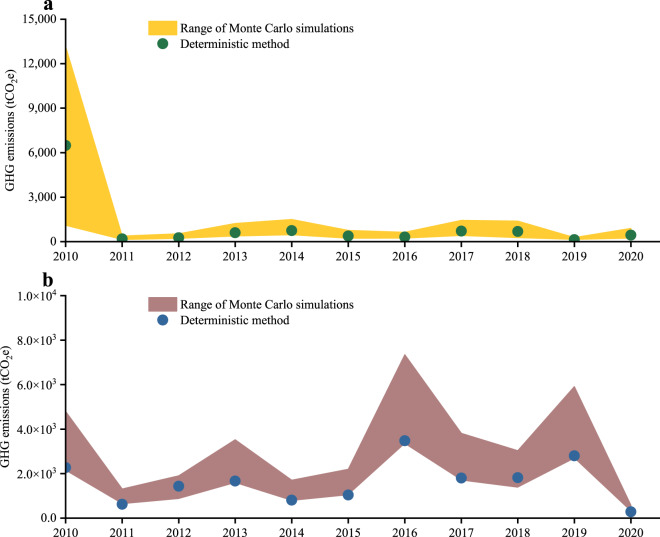
Results comparison between Monte Carlo simulation and deterministic methods. (**a**) Gathering pipelines. (**b**) Transmission pipelines.

Figure 5 and Supplementary Tables 4, 5 indicate that the GHG emission estimates obtained through deterministic methods fall within the upper and lower bounds of the Monte Carlo simulation. The estimated biases for gathering pipelines range from −1.37% to −0.90%, while the estimated biases for the transmission pipeline range from −5.64% to 33.71%. It demonstrates the high reliability of the proposed GHG emission estimation method and the generated datasets in this study. Furthermore, after 2010, there has been a decrease in GHG emissions resulting from crude oil pipeline accidents. It is primarily attributed to an improved response speed to accidents and implementation of effective emergency measures and rapid repairs^44^, leading to reduced duration and smaller scale of leaks after accidents, and thus, lower GHG emissions.

### Supplementary information

Supplementary Information

## Data Availability

The code utilized for the Monte Carlo simulations in this study is provided in the Supplementary Codes 1 and 2.
