# Supplementary material for: Greenhouse gas emissions from U.S. crude oil pipeline accidents: 1968 to 2020
Source: Sci Data. 2023 Aug 24;10:563. doi: 10.1038/s41597-023-02478-4 (PMC10450021; doi:10.1038/s41597-023-02478-4)
Supplement: Supplementary file 1 — Supplementary Information [file 41597_2023_2478_MOESM1_ESM.docx]

**Supplementary Information**

*Greenhouse gas emissions from U.S. crude oil pipeline accidents: 1968 to 2020*

*Lu et al.*

(a)

(b)

**Supplementary Fig. 1. State-level** GHG emissions arising from crude oil pipeline accidents in the U.S. from 1968 to 2020. The displayed values represent the mean of the Monte Carlo simulation results. White represents unavailable data for the state, indicating that there were no crude oil pipeline spills in the state between 1968 and 2020. The offshore area is for illustrative purposes only and does not represent the actual boundary of the area.

**Supplementary Table 1.** Primary differences between different oil pipelines^S1^.

| **Oil pipeline category** | Crude oil gathering pipelines | Crude oil transmission pipelines |
| --- | --- | --- |
| **Function** | Collecting crude oil from well site to processing plants | Transporting crude oil to remote refineries |
| **Media** | Crude oil, natural gas, and water | Crude oil and minute quantity of natural gas |
| **Volume of GHGs escaped after pipeline leaks** | Typically large volume | Minute quantity |
| **Conditions for generating GHG emissions** | Leakage, combustion, and explosion | Leakage, combustion, and explosion |
| **Distance** | Typically, the length of a single gathering pipeline ranges from several hundred meters to several kilometers. | The length of a single transmission pipeline can reach thousands of kilometers. |
| **Remark** | Due to the presence of unrecovered natural gas within the pipelines, a substantial volume of natural gas is usually released from crude oil flows. | The natural gas is removed after processing, leaving only a small amount of gas dissolved in the crude oil, which is subsequently released when pressure rapidly decreases. |

**Supplementary Table 2.** Data sources involved in this study.

| Data | Data sources | Remarks |
| --- | --- | --- |
| Oil pipeline accidents | PHMSA website (https://www.phmsa.dot.gov/data-and-statistics/pipeline/distribution-transmission-gathering-lng-and-liquid-accident-and-incident-data#:~:text=Incident%20reporting%20requirements%20%20%20Pipeline%20System%20Type,CFR%20191.3%20%20%2049%20CFR%20191.15) |  |
| Production Gas-Oil Ratio (PGOR) | https://www.eia.gov/petroleum/wells/ | The PGOR is calculated based on the data collected from a total of 12,414 wells from 2000 to 2021, as provided in this dataset. |
| Natural gas specific gravity | Dembicki, H. (2022). Practical petroleum geochemistry for exploration and production. Elsevier. | / |
| API value | EIA (https://www.eia.gov/dnav/pet/pet_crd_api_adc_mbblpd_m.htm) | A total of 2,290 data points were collected from January 1985 to February 2023. |
| GWP value | IPCC website (https://www.ipcc.ch/report/ar6/wg1/downloads/report/IPCC_AR6_WGI_Chapter_07_Supplementary_Material.pdf) | / |

**Supplementary Table 3.** Data range of parameters in Monte Carlo simulation and values in deterministic methods.

| Data | Data range in Monte Carlo simulation | Values in deterministic methods |
| --- | --- | --- |
| PGOR | 1.0-5981.9 ft^3^/bbl | 935 ft^3^/bbl (average value of 12,414 data) |
| Natural gas specific gravity | 0.55-0.87 | 0.71 |
| API value | 23.99-37.90 | 31.70 |
| $f_{u}$ | 0-1 | 0.5 |
| $f_{c}$ | 96%-100% | 98% |

**Supplementary Table 4.** Deviation between Monte Carlo simulations and deterministic method for gathering pipelines from 2010 to 2020.

| Year | Average of Monte Carlo simulation | Deterministic method | Deviation *E* |
| --- | --- | --- | --- |
| 2010 | 6578 | 6488.718445 | -1.37% |
| 2011 | 191 | 189.4860752 | -0.96% |
| 2012 | 265 | 262.4680154 | -1.08% |
| 2013 | 612 | 605.0454289 | -1.07% |
| 2014 | 751 | 743.6893852 | -1.03% |
| 2015 | 381 | 376.9978198 | -1.01% |
| 2016 | 318 | 314.9678271 | -1.11% |
| 2017 | 718 | 711.2743525 | -0.90% |
| 2018 | 693 | 684.763686 | -1.20% |
| 2019 | 139 | 137.2842757 | -1.06% |
| 2020 | 448 | 443.5414553 | -1.05% |

**Supplementary Table 5.** Deviation between Monte Carlo simulations and deterministic method for transmission pipelines from 2010 to 2020.

| Year | Average of Monte Carlo simulation | Deterministic method | Deviation *E* |
| --- | --- | --- | --- |
| 2010 | 2397 | 2270.033294 | -5.59% |
| 2011 | 656 | 621.4296563 | -5.56% |
| 2012 | 952 | 1436.085465 | 33.71% |
| 2013 | 1761 | 1666.96616 | -5.64% |
| 2014 | 854 | 808.4883977 | -5.63% |
| 2015 | 1097 | 1041.461839 | -5.33% |
| 2016 | 3672 | 3476.415433 | -5.63% |
| 2017 | 1905 | 1803.487776 | -5.63% |
| 2018 | 1515 | 1820.740366 | 16.79% |
| 2019 | 2956 | 2799.40203 | -5.59% |
| 2020 | 292 | 283.5896743 | -2.97% |

**Supplementary Note 1: Definition of crude oil pipeline accidents**

An accident report is obligatory for each instance of failure in a pipeline system falling within the scope of this regulation, where there occurs a release of crude oil resulting in any of the subsequent scenarios^S2^:

(a) Non-deliberate explosion or fire caused by factors other than operator intent.

(b) Discharge of 5 gallons (19 liters) or more of crude oil, with the exception that no report is required for a release of less than 5 barrels (0.8 cubic meters) resulting from routine pipeline maintenance activities.

(c) Fatality of any individual.

(d) Personal injury necessitating hospitalization.

(e) Estimated property damage, encompassing the expenses incurred for clean-up and recovery, valuation of lost product, and harm to the property of the operator or third parties, or both, surpassing $50,000.

**Supplementary Code 1: Python code of Monte Carlo simulation for estimating GHG emissions of gathering pipelines**

import numpy as np

import numpy

import random

import xlrd

import openpyxl

workbook=xlrd.open_workbook(r'Gathering pipeline simulation.xlsx')

sheet=workbook.sheet_by_index(0)

row = sheet.nrows

col = sheet.ncols

aaa=np.zeros((row,col))

for i in range(row):

for j in range(col):

aaa[i][j]=sheet.cell(i,j).value

workbook1=openpyxl.Workbook()

booksheet1=workbook1.create_sheet('Gathering')

for k in range(200000):

for j in range(col):

p=5980*random.betavariate(1.02, 5.48)+1

while p<0:

p=5980*random.betavariate(1.02, 5.48)+1

booksheet1.cell(k+1,j+1).value=aaa[0][j]*p*np.random.uniform(0.66,1.05)*0.000028*27.9

workbook1.save('Gathering pipeline.xlsx')

**Supplementary Code 2: Python code of Monte Carlo simulation for estimating GHG emissions of transmission pipelines**

import numpy as np

import numpy

import random

import xlrd

import openpyxl

workbook=xlrd.open_workbook(r'transmission pipeline simulation.xlsx')

workbook0=openpyxl.Workbook()

booksheet0=workbook0.create_sheet('transmission')

sheet=workbook.sheet_by_index(0)

row = sheet.nrows

col = sheet.ncols

aaa=np.zeros((row-1,col))

for k in range(200000):

for i in range(row-1):

aaa[i][0]=sheet.cell(i+1,0).value

aaa[i][1]=sheet.cell(i+1,1).value

aaa[i][3]=sheet.cell(i+1,3).value

if sheet.cell(i+1,4).value=='Yes':

if type(sheet.cell(i+1,2).value)==type('string'):

aaa[i][2]=random.betavariate(0.505, 0.0561)

while aaa[i][2]<0 or aaa[i][2]>1:

aaa[i][2]=random.betavariate(0.505, 0.0561)

elif sheet.cell(i+1,2).value==0:

aaa[i][2]=1

elif sheet.cell(i+1,4).value=='No':

if type(sheet.cell(i+1,2).value)==type('string'):

aaa[i][2]=0

else:

aaa[i][2]=sheet.cell(i+1,2).value

Rs=np.random.uniform(0.55,0.87)*(aaa[i][3]/18*10**(0.0125*(23+random.weibullvariate(10.8,6.35)))/10**(0.00091*60))**(1.2048)

aaa[i][4]=aaa[i][1]*Rs

if sheet.cell(i+1,4).value=='No':

booksheet0.cell(k+1,i+1).value=aaa[i][4]*0.000028*27.9*np.random.uniform(0.66,1.05)

elif sheet.cell(i+1,4).value=='Yes':

booksheet0.cell(k+1,i+1).value=aaa[i][4]*0.0000548*np.random.uniform(0.96,1)+aaa[i][2]*aaa[i][1]*0.43

workbook0.save('Transmission pipeline.xlsx')

**Reference**

S1. Lu, H., Behbahani, S., Azimi, M., Matthews, J. C., Han, S., & Iseley, T. Trenchless construction technologies for oil and gas pipelines: State-of-the-art review. *J. Constr. Eng. M*. **146**, 03120001 (2020).

S2. US Government Publishing Oﬃce. Electronic Code of Federal Regulations. https://www.ecfr.gov (2023).
